# Brain Processing of Emotional Scenes in Aging: Effect of Arousal and Affective Context

**DOI:** 10.1371/journal.pone.0099523

**Published:** 2014-06-16

**Authors:** Nicolas Gilles Mathieu, Edouard Gentaz, Sylvain Harquel, Laurent Vercueil, Alan Chauvin, Stéphane Bonnet, Aurélie Campagne

**Affiliations:** 1 Laboratoire de Psychologie et Neurocognition (LPNC), Univ. Grenoble Alpes, Grenoble, France; 2 LPNC, Centre National de la Recherche Scientifique, Grenoble, France; 3 Faculté de Psychologie et des Sciences de l'Education, Université de Genève, Switzerland; 4 IRMaGe, Univ. Grenoble Alpes, Grenoble, France; 5 Unité Mixte de Recherche 3552 (UMR3552), Centre National de Recherche Scientifique, Grenoble, France; 6 Unité de Service 17 (US17), Institut National de la Santé et de la Recherche Médicale, Grenoble, France; 7 IRMaGe, Centre Hospitalier Universitaire de Grenoble, Grenoble, France; 8 Service Exploration Fonctionnelle du Système Nerveux (SEFSN), Univ. Grenoble Alpes, Centre Hospitalier Universitaire de Grenoble, Grenoble, France; 9 Grenoble Institut of Neurosciences (GIN), Univ.Grenoble Alpes, Grenoble, France; 10 GIN, Institut National de la Santé et de la Recherche Médicale, Grenoble, France; 11 GIN, Centre Hospitalier Universitaire de Grenoble, Grenoble, France; 12 Laboratoire d'Électronique de Technologie de l'Information (Leti), Univ. Grenoble Alpes, Grenoble, France; 13 Leti, Commissariat à l'énergie atomique et aux Energies Alternatives, MINATEC Campus, Grenoble, France; Centre de Neuroscience Cognitive, France

## Abstract

Research on emotion showed an increase, with age, in prevalence of positive information relative to negative ones. This effect is called positivity effect. From the cerebral analysis of the Late Positive Potential (LPP), sensitive to attention, our study investigated to which extent the arousal level of negative scenes is differently processed between young and older adults and, to which extent the arousal level of negative scenes, depending on its value, may contextually modulate the cerebral processing of positive (and neutral) scenes and favor the observation of a positivity effect with age. With this aim, two negative scene groups characterized by two distinct arousal levels (high and low) were displayed into two separate experimental blocks in which were included positive and neutral pictures. The two blocks only differed by their negative pictures across participants, as to create two negative global contexts for the processing of the positive and neutral pictures. The results show that the relative processing of different arousal levels of negative stimuli, reflected by LPP, appears similar between the two age groups. However, a lower activity for negative stimuli is observed with the older group for both tested arousal levels. The processing of positive information seems to be preserved with age and is also not contextually impacted by negative stimuli in both younger and older adults. For neutral stimuli, a significantly reduced activity is observed for older adults in the contextual block of low-arousal negative stimuli. Globally, our study reveals that the positivity effect is mainly due to a modulation, with age, in processing of negative stimuli, regardless of their arousal level. It also suggests that processing of neutral stimuli may be modulated with age, depending on negative context in which they are presented to. These age-related effects could contribute to justify the differences in emotional preference with age.

## Introduction

A large number of behavioral [1], [2] and neuroimaging studies [3]–[5] showed that negative stimuli engage more attention and induce more brain activity in regions involved in emotional and visual processing, relative to positive or neutral stimuli.

In normal aging, the preference for the negative stimuli (named negativity bias) tends to disappear and, sometimes, to shifts towards a preference for the positive stimuli compared to both the negative and neutral stimuli (named positivity bias). This modulation in emotional preference with age is called the positivity effect and could be mainly related to a decrease in processing of negative information and in attention to this type of information. Only a few studies rather suggest an improvement in processing of positive information with age [6]–[8].

Various theories tend to explain this age effect [9]–[11]. According to the Socioemotional Selectivity Theory (SST) [11]–[13], the reduction of older people's live span could motivate them in giving priority to their well-being and to favor positive information over negative information. This age-related positivity effect constitutes a strategy of mood regulation [14], implemented by cognitive control mechanisms, which evolve with older adults' motivations. These control processes could modulate the salience of negative information and/or positive information and could contribute to the positivity effect seen with age [15], [16]. The aging brain model [9], another theory, supposes an atrophy of cerebral structures involved in emotional processing, such as the amygdala. This atrophy would alter the processing of negative stimuli and could lead to the positivity effect.

The positivity effect is mainly reported in behavioral studies. In recall memory tasks, healthy older people remember more often positive stimuli than negative ones (pictures or faces) compared to younger adults [7], [17]–[19]. The positivity effect is also shown in attention paradigms [2]. For instance, during the passive viewing tasks of two emotional stimuli (scenes or faces) both displayed simultaneously, the gaze fixation is typically longer for positive stimuli than for negative stimuli in older adults, this compared to young adults [20]–[22]. In a dot-probe task, Mather and Carstensen [23] also show that older adults are attracted to the positive stimuli and avoid the negative stimuli. Various fMRI studies mainly highlight an age effect on processing of negative stimuli [15], [24], in several regions of the brain such as the amygdala and the perfrontal cortex. The amygdala would particularly contribute to the evaluation of the emotional salience of stimuli and the prefrontal cortex would be involved in emotional regulation [25]–[29]. Specifically, during the processing of negative pictures [25], [27], [29], older adults show lower amygdala activities and higher prefrontal activities compared to younger adults. The positivity effect is also observed in the few ERP studies performed on elderly. Specifically, the amplitude of the Late Positive Potential (LPP) evoked by negative stimuli, decreases in older adults and becomes similar to the LPP amplitude evoked by positive stimuli [30]–[32]. According to literature, the amplitude of this slow wave component, peaking around 300–900 ms in centro-parietal region [33], would reflect the attentional engagement [34], [35] and would be sensitive to emotional regulation processes [36].

Some behavioral studies based on emotional appraisal tasks [37], [38], or memory or attention tasks [19], [39], suggest that the arousal level of stimuli may modulate the age effect on emotional information processing [19], [38], [40]. Streubel and Kunzmann [38] show that the low-arousal negative stimuli are rated as less unpleasant in older adults compared to younger adults. Other authors show the older adults rate the high-arousal negative stimuli as more unpleasant [37], this compared to younger adults. For positive stimuli, the low-arousal stimuli are rated as more pleasant [38]; in contrast, variable results are observed for the high-arousal positive stimuli [37], [38]. Kensinger [19] shows that, in young and older adults, the negative words are recalled more often than the positive words, for the high-arousal words. Whereas the opposite pattern is observed for the low-arousal words, in older adults. Mather and Knight [39] also observe that the detection of threatening stimuli, with a high-arousal level, is similar between older and younger adults. Taken together, these data thus suggest that processing of high-arousal information, which mainly involves automatic process [41], remains relatively well preserved with age, particularly for the negative stimuli [39], [41]. In contrast, it is assumed that the positivity effect is due to a controlled, rather than an automatic processes [20], [42]. Processes which would be particularly favored during the processing of low-arousal stimuli. These assumptions support the SAVI model (Strength and Vulnerability Integration) [43]. In the continuity of the “cognitive control” model, the SAVI model assumes that emotional regulation processes, in older adults, would be facilitated, and most efficiently, during the processing of low-arousal information. In contrast, in older adults, the processing of high-arousal information could limit the influence of emotional regulation processes (processes which are demanding in resources), because of a decreased flexibility of the physiological system, due to aging i.e. the internal homeostatic balance is restored later in older adults compared to younger adults [37], [43]. However, these assumptions are based on behavioral studies and the effect of arousal level on cerebral processing of visual emotional information with age, has not yet been studied.

In addition, studies showed that stimuli are automatically evaluated in their environment [44], i.e. by taking into account the others information simultaneously or previously displayed. In an ERP study, Smith and collaborators [45] show that the processing of negative stimuli (pictures) may be favored, or be attenuated as a function of the valence and the probability of preceding stimuli. According to another ERP study, using an affective priming paradigm [46], it appears that the affective context effect also depends on arousal level of emotional stimuli and may be favored during high-arousal stimuli processing, regardless of the stimuli valence.

Few studies explored the influence of affective context on processing of emotional stimuli with age and only in mood induction paradigms. Isaacowitz et al. [47] observe that, when their mood is negative, older adults do not focus their attention on negative stimuli but rather on positive stimuli (positivity effect), whereas younger adults show an opposite pattern based on the same mood. The SAVI model assumes that following an emotion, the internal homeostatic balance is restored later in older adults, compared to younger adults. Therefore, we assume that affective context effect on processing of emotional stimuli would be amplified with age. According to the SAVI model, the perturbation in homeostatic balance with age would disturb cognitive processes such as emotional regulation processes (which are demanding in resources), this, particularly in high-arousal conditions, rather than in low-arousal conditions. If the positivity effect is mainly related to emotional regulation processes, as suggested by cognitive control theories (SST and SAVI models), it could be favored in a low-arousal context i.e. when the regulation processes are less disturbed by the affective context. However, to our knowledge, the contextual effect of arousal level on cerebral processing of emotional stimuli with age has not yet been examined.

In the present event-related potentials (ERP) study, we investigated to which extent two arousal levels of negative scenes (high-arousal and low-arousal) are differently processed with age and, to which extent these different arousal levels of negative stimuli may contextually modulate the cerebral processing of positive (and neutral) scenes and contribute to the observation of a positivity effect with age, i.e. reduction of the preference for negative information in older adults, compared to younger adults, in favor of positive stimuli. In this aim, two negative picture groups, characterized by two distinct arousal levels, were selected and displayed into two separate experimental blocks, in which were included positive and neutral pictures. The two blocks only differed by their negative stimuli, as to create two negative contextual blocks for the processing of positive (and neutral) stimuli. Pictures were presented in a repeated pseudo-random sequence, as to induce a negative context substantial and homogeneous throughout the block. Because of the prevalence of these effects in literature [2], we focused our study on age-related effects on negative stimuli and their consequences on processing of positive (and neutral) stimuli. Our evaluation was performed from cerebral analysis of the LPP amplitude. The LPP amplitude is sensitive to both arousal level [48] and emotional regulation processes [36] and constitutes a relevant indicator of the positivity effect [30], [31].

We expected the processing of high-arousal negative stimuli to be preserved with age, contrary to low-arousal negative stimuli. Specifically, we assumed that the LPP amplitude induced by the high-arousal negative stimuli would be relatively similar in the two age groups, whereas the LPP amplitude induced by the low-arousal negative stimuli would be decreased in older adults. Although literature do not show strong age effects on processing of positive stimuli, we also hypothesized that the LPP amplitude of positive stimuli could be increased with age, and most important, in the contextual block of low-arousal negative stimuli. Based on these hypotheses, these age-related effects could contribute to justify differences in emotional preference with age.

## Methods

### Ethics statement

The study was promoted by the University Hospital of Grenoble, approved by the local French ethics committee (Comité de protection des personnes Sud-Est V, ID RCB: 2011-A01602-39) and French health safety agency (B111591-30) and conducted according to the principles expressed in the Helsinki Declaration. All participants signed an informed consent and received a 40-euro indemnity.

### Participants

Fourteen young adults (7 men; 24.4 *SE*±1.8 years; 7 women; 27.5 *SE*±1.4 years) and fourteen older adults (7 men; 62.3 *SE*±0.8 years; 7 women; 65.3±*SE* 1.6 years) participated in the experiment. The two age groups had a French culture and a similar education level and all participants were right-handed. They were selected from 19 young adults and 23 older people following a medical and neuropsychological screening. They had a normal or corrected-to-normal vision. They were free of any medical treatment likely to modulate emotional processes (e.g. beta-blocking) or any neurological or psychiatric disorder, past or present. Each participant had a minimal score of 27 in the Mini Mental State Examination (MMSE), a maximum of 4 on the short version of the beck depression scale and made no more than 12 errors during the short version of the Wisconsin Card Test.

### Stimuli

Stimuli were displayed against a black background using E-prime software (E-prime Psychology Software Tolls Inc., Pittsburgh, USA) onto a 22-inch monitor located 90 cm from the participants. They consisted of 180 color pictures of natural scenes (at a resolution of 640×480 pixels) projected with a visual angle of 16.4°×11.4°. Stimuli were composed of 60 negative, 60 positive and 60 neutral scenes. Visual scenes were in close-up and directly involved the participant. Pictures were selected from several sources: Internet (not copyrighted) and an in-house database and the International Affective Picture System (IAPS, numbers: 1050, 1052, 1300, 1303, 2811, 1560, 1650, 2692, 5940, 6250, 6260, 6510, 7009, 7161) [49]. Negative scenes included dangerous animals (spiders, snakes, sharks, etc.), unsafe environments (tornadoes, fires, tsunamis, etc.) and aggressive people (carrying weapons, angry expressions, etc.). Positive scenes included images showing safe, happy and friendly animals, idyllic landscapes (beaches, mountains, etc.) as well as happy and friendly people. Neutral scenes included the same type of stimuli (animals, environments, objects and people) in neutral situations. Pictures are available from the corresponding authors.

Based on a pretest performed with 34 young volunteers (22.7 years old *SE*±0.21, 20 female, 14 male), the 180 pictures were categorized and selected (from 420 pictures) depending on their discrete emotion and their arousal level (evaluated on a continuous arousal scale from 1-none to 9-high), which were variables of interest in our study. It has to be noted that we verified, during the experiment, the validity of these appraisals (discrete emotion and arousal level) in young participants, and assessed the possible modulations with age (see section protocol). The 180 pictures were also re-rated in terms of valence (evaluated on a continuous valence scale from 1-most unpleasant to 9-most pleasant) during another control test, with 19 young adults (21.6 years old SE±0.3 years) and 17 older adults (65 years old SE±0.9 years), which were selected according to the same procedure as used for the main experiment. According to an ANOVA with age group (young and older adults) as a between-subjects factor and emotional stimuli category (categories defined below) as a within-subjects factor, the mean valence ratings reported for each category was not statistically different between our two age groups. Negative stimuli were associated to a fear emotion and were subdivided into two groups depending on their arousal level, i.e. 30 low-arousal negative stimuli (4.12 SE±0.6) and 30 high-arousal negative stimuli (7.48 SE±0.35). The mean valence of negative stimuli was 1.9 (young adults: 1.82 SE ±0.15; older adults: 2.01 SE ±0.12) for high-arousal negative stimuli, and 3.03 (young adults: 2.87 SE±0.16; older adults: 3.2 SE ±0.14) for the low-arousal negative stimuli. Positive stimuli were characterized by a mean valence of 7.07 (young adults: 7.12 SE ±0.16; older adults: 7.02 SE ±0.18), an emotion of well-being (i.e. pleasure, joy) and a mid-arousal level (5.38 SE±0.8). Neutral scenes without emotional content were defined by a mean valence of 4.82 (young adults: 4.71 SE ±0.09; older adults: 4.95 SE ±0.13), a very low-arousal level (1.2 SE±0.2). The luminosity and contrasts were corrected to be similar between the 4 categories of pictures. The picture ratings by age group are available in the Table S1.

### Protocol

Brain activity of the two age groups was recorded during an emotional categorization task of natural scenes. Two 8-minute experimental blocks were performed by participants with breaks in between. Each block included 30 negative pictures, 30 positive pictures and 30 neutral pictures. The two blocks only differed by the arousal level of their negative images - one block included high-arousal negative pictures and the second block included low-arousal negative pictures. Between blocks, two groups of positive and neutral stimuli were used, in order to avoid an habituation effect. However, these two stimuli groups had the same emotional characteristics and were counterbalanced between blocks across participants, in each age group (so as to have equivalent experimental blocks by their positive and neutral stimuli across all participants in each group). These blocks were defined so as to specifically assess the contextual influence of two arousal levels of the negative stimuli on processing of positive and neutral stimuli. In each block, the negative stimuli were displayed among the positive and neutral stimuli, according to a pseudo-random sequence of two stimuli of each valence which was repeated 15 times (with different stimuli for each valence), so as to induce a negative context substantial and homogeneous throughout the block. The context effect that we wanted to study here was a “global context” effect of negative stimuli on processing of positive and neutral stimuli on the entire block considered. Given the two blocks only differed by the nature of the negative stimuli across participants of each age group, the differences that we may observe in processing of positive (and neutral) stimuli between the two blocks will be associated to the nature of the negative stimuli in each block. In order to avoid any experimental bias, the block order was randomly distributed across participants of the same age group. Each trial began with a central fixation cross for a variable period (1500 ms±500 ms), followed by the 1000 ms display of the stimulus. At the end of each stimulus presentation, and for 300 ms, a white dot was flashed upon the screen to indicate the time for the participants to answer. The response-time period was 2000 ms. For each trial, the participants were instructed to view and consider the scene as if it was real. They had to report their emotional experiences (fear for their physical integrity, well-being or no emotion) as spontaneously as possible by pressing one of the three corresponding keyboard keys by using their right hand. Before the 2 experimental blocks, 30 different training trials from those used in the experiment, were projected to familiarize the participant with the task ahead.

Following the experiment, the arousal level induced by each image used in the experiment, was re-evaluated by young and older participants on a continuous scale from 1- none to 9-high by using a joystick (following the same procedure as our pre-test). This, in order to assess the possible appraisal difference with age (Table S1). For that, the 180 pictures were again displayed for 1 s in a random sequence.

It should be noted that, in literature, others paradigms have been more commonly used to assess the effect of affective context. We did not use an oddball paradigm in order to avoid probability effects and task-related selective attention effects on cerebral activity that change with age, independently of the emotional context effects [50]. The use of a priming paradigm also seemed present methodological problematic. Indeed, it is known that priming effects depend on delay between prime and target (Stimulus Onset Asynchrony, SOA; [51]). Moreover, various studies show that the speed of brain processing of information is slower in the elderly compared to young people [52]. It was, therefore, difficult to determine an optimal SOA for the two populations (young and older adults) tested in our study. Also, we did not use a classic emotional induction paradigm. We wanted to further assess the processing influence of an emotional information kind (negative) on the processing of other emotional information kinds (positive and neutral) within the same presentation sequence, rather than the influence of an emotional state (or a mood) induced prior to the presentation of stimuli. Finally, we consider the paradigm chosen to be more ecological, i.e. the manipulation of context in the study is less transparent to subjects and minimizes demand biased responses [53].

### Data recordings and analysis

#### Behavioral and subjective data

For each participant, the accuracy, as the mean rate of correct responses (%ACC), were measured for each stimulus category, in each experimental condition. A response for a given scene was considered correct when the response (emotion) given by the participant was the same as the one given by the pretested participants, i.e. when the response is congruent. Specifically, a correct response corresponded to: “press the fear response key” for the pictures characterized by a fear emotion during the pretest; and “press the well-being response key” for the pictures characterized by a well-being emotion during the pretest; and “press the neutral response key” for the pictures categorized as neutral during the pretest. All different responses (i.e. non congruent and button errors) were considered errors. It should be noted that no significant differences appeared between our participants and our pretest group and were similar between the age groups. The mean rate of correct responses was assessed using a repeated-measure ANOVA with participants' age (young and older adults) as a between-subjects factor and contextual block (high arousal or low arousal context) and emotion (negative, positive, neutral) as within-subjects factors.

The mean subjective arousal level reported by each participant after the EEG experiment was studied depending on age (between-subject factor) and the 4 emotional stimuli categories (high-arousal negative, low-arousal negative, positive and neutral; within-subject factor) by using a repeated-measure ANOVA.

Mean comparisons of behavioral and subjective data were performed using Tukey post hoc. The significant level of tests was set at 0.05.

#### EEG data

The electroencephalographic (EEG) activity was continuously recorded during the two experimental blocks by using an Acticap (Brain Products, Inc.) equipped with 78 Ag-AgCl unipolar active electrodes which were positioned according to the extended 10–20 system [54], [55]. The reference and ground electrodes used for EEG data acquisition were those of Acticap, i.e. close to the vertex (FCz) for the reference electrode, and on the forehead (AFz) for the ground electrode. For the other electrodes, a ground electrode was placed on the shoulder blade. Blinks and eye movements were recorded using 4 electro-oculography electrodes: two positioned above and below the left eye on the median axis for vertical activities and two at the two each corner of the eyes. In addition, participants were instructed to avoid blinking or to move their eyes, both during the fixation cross and the picture presentation. The signals' impedance was below 5 kΩ. Data were amplified using a BrainAmp system (Brain Products, Inc.) and sampled to 1000 Hz, with a 0.1 µV resolution and a 10-second time constant. The acquisition of data was performed in Grenoble EEG facility IRMaGE.

Using BrainAnalyzer software, the EEG raw data were digitally filtered by applying a low-pass band below 30 Hz. The EEG data were segmented into epochs from 350 ms before stimulus onset to 1000 ms after stimulus onset. Each segment was reviewed offline and all segments containing ocular blink, muscular activity or non-physiological artifacts were rejected. Artifacts related to ocular movements were corrected by using an independent component analysis (ICA). Data were then baseline corrected to the 350 to 50 ms preceding the stimulus. For each participant, from the two age groups, Event Related Potentials (ERPs) induced by pictures and measured over each electrode, were averaged in each block, depending on the emotional category of stimuli: high-arousal negative stimuli (n = 27.6 trials ±1.75), positive stimuli (n = 27.5 trials ±2) and neutral stimuli (27.6 trials ±1.7) for one block, and low-arousal negative stimuli (n = 27.6±1.63), positive stimuli (n = 27.5 trials ±1.97) and neutral stimuli (n = 27.4 trials ±1.97) for the other block.

Our analysis focused here, on Late Positive Potential (LPP) which we identified based on the grand average of data, in each age group. The LPP was observed between 400 and 700 ms from stimulus onset [56]–[59], in the centro-parietal region. It was more specifically measured at the amplitude peak of the component, i.e. Pz, in accordance with the literature data [3], [30], [31], [33]. For each participant, the mean amplitude of the LPP was measured over the Pz electrode, for each emotional valence, in the two experimental blocks. The LPP amplitude was then analyzed using an ANOVA for repeated measures with participants' age (young and older adults) as a between-subjects factor and contextual block (contextual block with high or low arousal negative stimuli) and emotion (negative, positive and neutral) as within-subjects factors. In the case of a sphericity violation in our analyses, a Greenhouse-Geisser correction was performed. Mean comparisons were explored for interactions showing a significant effect, or a tendency to significance, using planned contrasts or Tuckey post-hoc according to our hypotheses. Details are given in results. The significant level of tests was set at 0.05. To determine whether the gender of the participants influenced processing of the emotional stimuli, we previously performed a repeated-measure ANOVA for each analysis, with participants' age and gender as between-subjects factors and contextual block and emotion as within-subjects factors. No main gender effect, or any interaction with other factors, was observed (all p>.05). The gender factor was thus subsequently removed from the analyses reported here. All statistical analyses were performed using Statistica10 (StatSoft, Inc.) software.

## Results

### Subjective arousal level

Due to acquisition problems, only 22 participants (11 younger and 11 older adults) were included in the analysis of subjective arousal level performed after the experiment. Statistical analysis showed that subjective arousal level only varied with Emotion (*F*(3.60) = 221; *p*<.001); no Age effect (*F*(1.20) = 2.33, *p*>.14) and no interaction effect with Age (*F*(3.60) = 1.35,*p>*.27) were observed. In accordance with the pretest, high-arousal negative pictures (7.04, *SE*±0.26 on scale of 1 to 9) and neutral pictures (1.1, *SE*±0.11) elicited the highest and lowest subjective arousal level respectively, in comparison with positive stimuli (*p*<.001 for the two comparisons) and low-arousal negative stimuli (*p*<.001 for the two comparisons). Also, subjective arousal level for positive pictures (5.54, *SE*±0.2) was also significantly higher than for low-arousal negatives pictures (4.73, *SE*±0.31, *p*<.001).

### Percentage of correct responses (congruent responses, % ACC)

Based on the 28 participants, a significant main effect on mean rate of correct responses was observed for Contextual block (*F*(1, 26) = 9.24, *p*<.006). Mean comparisons revealed a higher rate in the block containing high-arousal negative stimuli (89.64%±2.33%), relative to the block with low-arousal negative stimuli (84.96%±2.72%). No main effect was observed for Age (*F*(1.26) = 1.48, ns) and for Emotion (*F*(2.52) = 0.79, ns). A significant effect of the two-way interaction Contextual block x Emotion on LPP amplitude was observed (*F*(2, 52) = 27.67, *p*<.001). Specifically, during the contextual block of high-arousal negative stimuli, performance was better for negative stimuli (94.41%, *SE*±2.21%) than for positive (85%, *SE*±2.25%, *p*<.001) and neutral stimuli (89.53%, *SE*±1.80%, *p*<.001). The opposite pattern was observed during the contextual block of low-arousal negative stimuli: positive (87.86%, *SE*±1.54%, *p*<.001) and neutral stimuli (88.69%, *SE* ±1.93%, *p*<.001) induced better performance than negative stimuli (78.34%, *SE* ±3.10%). Performance for positive and neutral stimuli was similar, regardless of the contextual block. No interaction effect with Age was noticed.

### Mean LPP amplitude analysis

**Figure 1:**
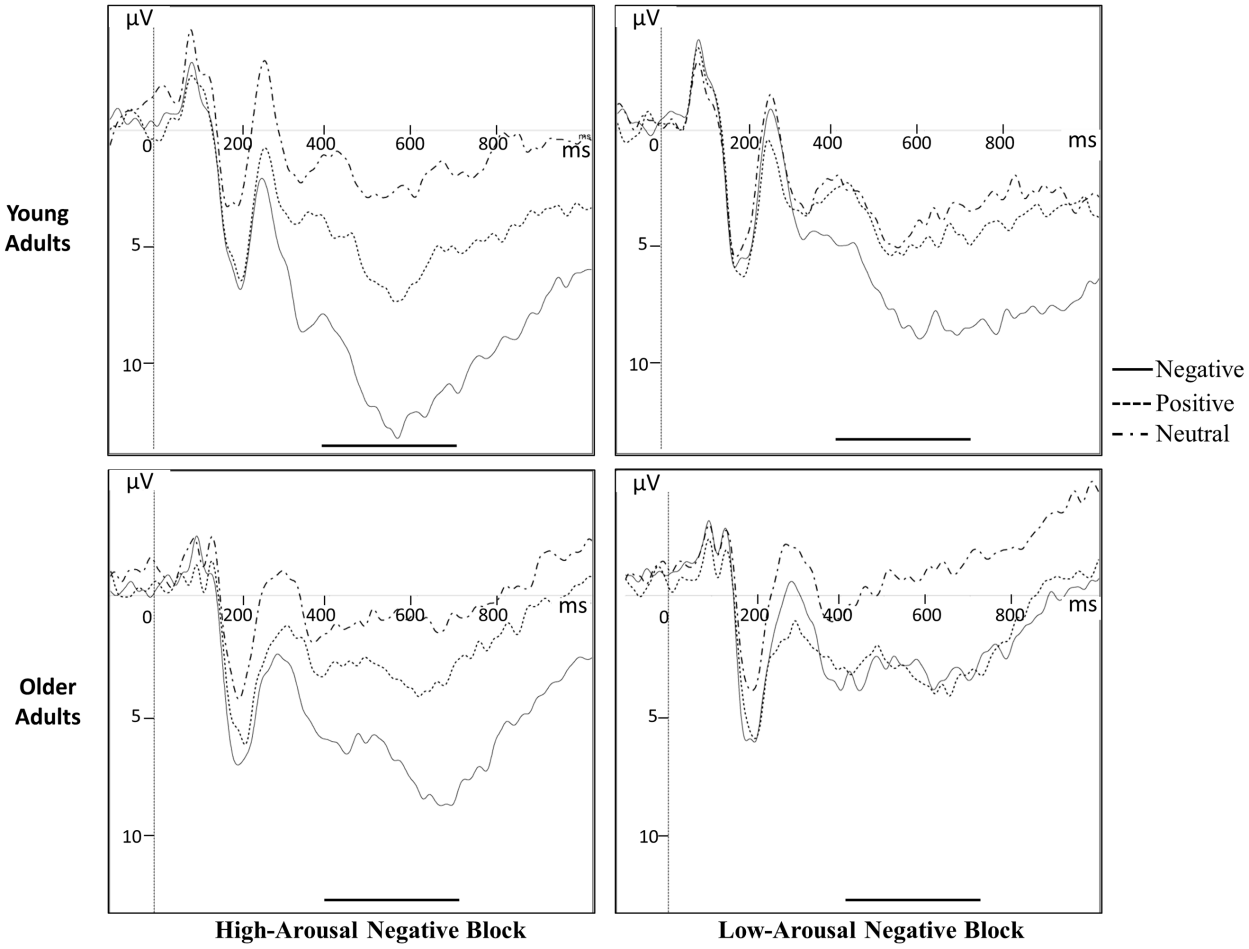
Group average of LPP at Pz electrode for the different experimental conditions in each age group. Positive values are oriented downwards. The continuous line, in bold, delimits the analysis time-window of LPP amplitude.

The behavioral data showed no statistical differences in emotional appraisals between younger and older adults. Therefore, we did not consider these appraisals as a co-variable in the ERP analysis of younger and older participants. Figure 1 shows the ERP waveform at Pz, for each emotion, in each contextual block of negative stimuli and for both age groups.

Based on the 28 participants, the ANOVA analyze revealed a main effect on LPP amplitude for Emotion (*F*(1.82, 47.3) = 67.60, *p*<.001) and a significant tendency for the two-way interaction Age x Emotion (F(1.82, 47.3) = 2.89, *p*<.065). No main effect of Age was observed (*F*(1, 26) = 2.74, *p* = 0.11). Planned comparisons performed for each emotion, between the two age groups, confirmed that the LPP amplitude for negative stimuli was statistically reduced in older adults compared to younger adults (*F*(1, 26) = 4.63, *p*<.041) and no significant difference between the two age groups was noted for neutral stimuli (*F*(1, 26) = 2.36, *p* = .14). Contrary to our expectations, no statistical increase of LPP amplitude was observed for positive stimuli with increasing age (*F*(1,26) = 0.95, *p* = .34).

**Figure 2:**
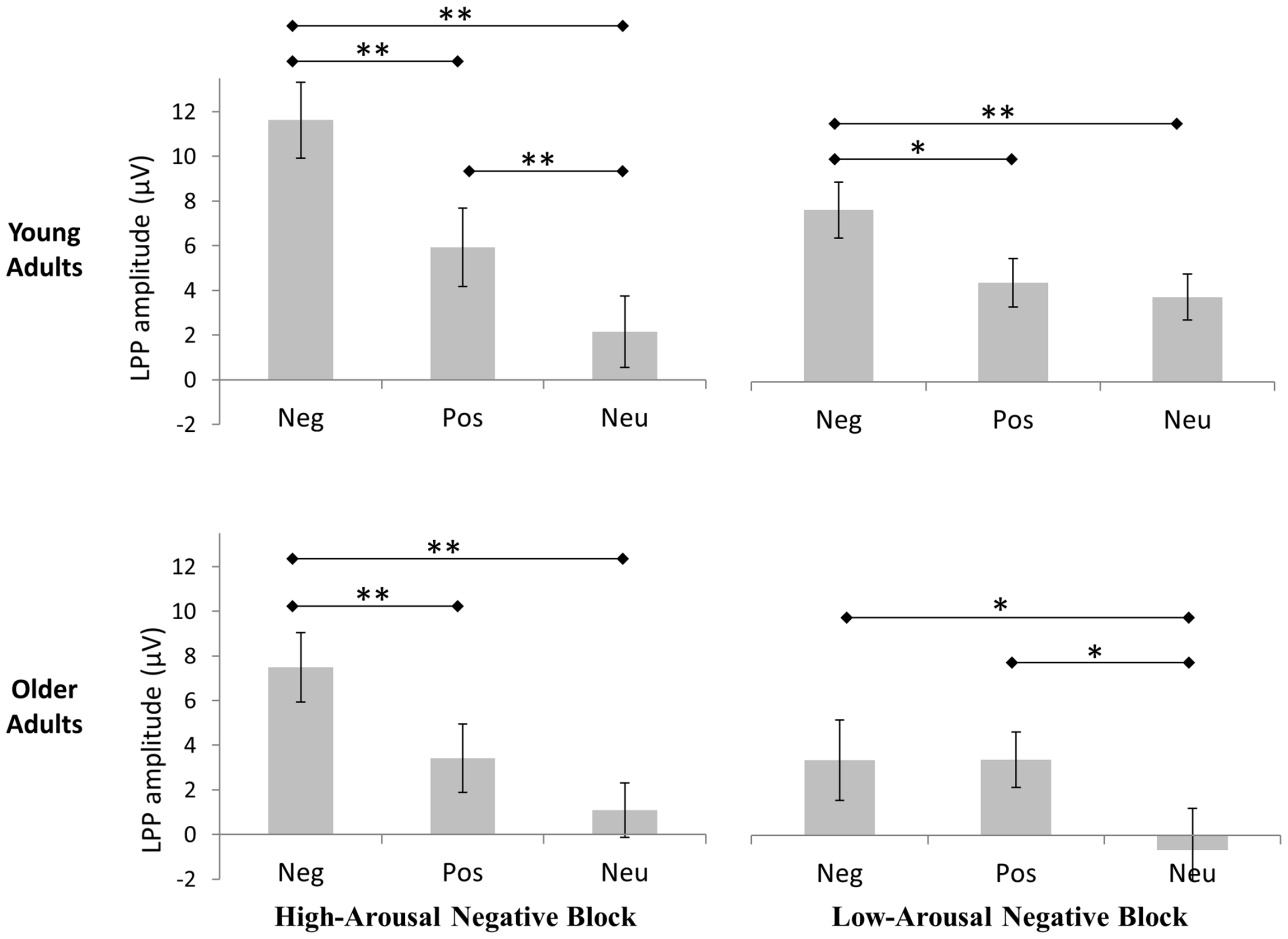
Mean amplitude of the LPP for the different experimental conditions in each age group. Significant post-hoc comparisons are represented by asterisks. ** 0.01 significant threshold, * 0.001 significant threshold.

However, we also showed that Contextual block (*F*(1, 26) = 6.22, *p*<.016) and the following interactions Contextual block x Emotion (*F*(1.73, 45) = 13.72, *p*<.001) and Age x Contextual block x Emotion (*F*(1.73,45) = 4.48, *p*<.022, η^2^ = .147; figures 1 and 2) significantly influenced the main LPP amplitude. The interaction Age x Contextual block was not significant (*F*(1,26) = .62, *p* = .63). For the 3-way interaction Age x Contextual block x Emotion, a post-hoc power analysis was performed using Gpower [60], [61]. Based on 14 participants per group, a significance threshold at .05, a non-sphericity correction ε at .0866 and a correlation coefficient between variables at 0 (the most conservative conditions), the power of the interaction is 0.89 to detect a population effect as large as that estimated here. This value is superior to the recommended value of 0.8 [62].

The two contextual blocks differed only by the arousal level of their negative pictures: one block included high-arousal negative pictures and the second block included low-arousal negative pictures. Planned comparisons performed for negative stimuli between the two contextual blocks, regardless of age group (in the significant interaction Contextual block x Emotion), showed that the low-arousal negative stimuli induced a lower LPP amplitude than the high-arousal negative stimuli (*F*(1,26) = 21.41, *p*<.001). These results physiologically confirm that the two types of negative stimuli used were distinct between the two blocks, independently of age group.

In addition, we assumed that the LPP amplitude induced by the high-arousal negative stimuli would be relatively similar in the two age groups, whereas the LPP amplitude induced by the low arousal negative stimuli would decrease in older adults compared to younger adults. From data of the significant 3-way interaction Age x Contextual block x Emotion, the analysis in planned comparison performed for negative stimuli between the two age groups and the two contextual blocks, did not confirm this hypothesis (*F*(1,26) = 0.005, *p* = .95).

In this study, we also wanted to estimate to which extent the arousal level of negative stimuli could contextually modulate the cerebral processing of positive and neutral scenes. On this issue, planned comparisons performed for positive stimuli between the two contextual blocks of negative stimuli, regardless of age group (in the significant interaction Contextual block x Emotion), revealed no significant LPP amplitude difference (*F*(1,26) = 1.58, *p* = .22). The same result was observed for neutral stimuli (*F*(1,26) = 0.008, *p* = .93).

We assumed that the contextual effect of negative stimuli on processing of positive and neutral stimuli depending on their arousal level, could vary with age. From data of the significant 3-way interaction Age x Contextual block x Emotion, planned comparisons performed for positive stimuli revealed no significant effect of the interaction Age x Contextual block (*F*(1,26) = 1.25, *p* = .27). In contrast, this interaction tended to be significant for neutral stimuli (*F*(1,26) = 3.5, *p*<.073). Specifically, means comparisons performed between the two age groups, for each contextual block, revealed that the LPP amplitude induced by the neutral stimuli was significantly higher for the younger adults compared to the older adults, in the contextual block of low-arousal negative stimuli (*F*(1,26) = 4.7, *p*<0.04) whereas, it was statistically similar between the two age groups in the contextual block of high-arousal negative stimuli (*F*(1,26) = 0.3, *p* = .59).

Finally, in order to estimate the emotional bias of each age group, in each contextual block of negative stimuli and this, without prior assumptions, the LPP amplitude of the three emotional categories were statistically compared, using a Tuckey post-hoc on data of the 3-way interaction Age x Contextual block x Emotion (Figures 1 and 2).

In the contextual block of high-arousal negative stimuli, young adults showed a significant higher LPP amplitude for negative stimuli compared to positive (*p*<.001) and neutral stimuli (*p*<.001), and for positive stimuli compared to neutral stimuli (*p*<.001). In the same context, we also noted in older adults, a significant higher LPP amplitude for negative stimuli, compared to positive (*p*<.001) and neutral stimuli (*p*<.001). No distinction was observed in older adults between the LPP amplitude induced by positive and neutral stimuli.

In the contextual block of low-arousal negative stimuli, young adults showed a significant higher LPP amplitude for negative stimuli compared to positive (p<.013) and neutral stimuli (*p*<.001), without significant difference between positive and neutral stimuli. In the same context, older adults showed a higher LPP amplitude for emotional stimuli, compared to neutral stimuli (*p*<.001 for the two comparisons), without significant difference between positive and negative stimuli.

## Discussion

Firstly, one of the main aims of this ERP study was to determine if the two arousal levels of negative scenes (high-arousal and low-arousal) were differently processed with age. The results revealed a classical decrease of the LPP amplitude, a reflect of arousal level and attention [34] for low-arousal negative stimuli compared to high-arousal negative stimuli. Contrary to our expectations, this reduction was independent of the participants' age, suggesting that the relative processing of different arousal levels of negative stimuli was performed on an equivalent scale between the two age groups. Nevertheless, negative stimuli induced lower LPP amplitude in older adults compared to young adults, independently of the arousal level of negative stimuli, suggesting an overall age-related reduction in processing and relevance of negative stimuli with increasing age. Such an age effect on processing of negative stimuli was reported in several neuroimaging studies [15], [27], [29] and in the few ERP studies performed on emotion in aging [30]–[32]. According to these studies and the main current theories (SST and aging brain model), this age-related effect on negative stimuli processing would be due i) to emotional regulation processes, which would decrease the salience of negative stimuli with age [15], and/or ii) to a possible cognitive decline with age that would degrade the negative stimuli [9].

In addition, we evaluated if the arousal level of the negative scenes could modulate the cerebral processing of positive (and neutral) scenes with increasing age. We assumed that age could favor the processing of positive stimuli, and most importantly, in the contextual block of low-arousal negative stimuli. In this context, we assumed that emotional regulation processes would be favored and more effective with age. Contrary to our expectations, no effect of age or context was observed in our study. In contrast, the cerebral processing of neutral stimuli was significantly reduced in older adults compared to younger ones, particularly in the contextual block of low-arousal negative stimuli, as attested by a lower LPP amplitude.

Taken together, all these data suggest that the processing of positive information is preserved with age and support the idea that age impacts the processing of negative information, in agreement with the meta-analysis performed by Murphy and Isaacowitz [2]. However, it appears that this age effect on processing of negative stimuli is independent of their arousal level. For stimuli negative, our results seem to refute the assumptions made in literature and model SAVI postulating a preserved processing with age for high-arousal information and not for low-arousal information. Interestingly, we show that age may also modulate the processing of neutral information, as suggested by some studies [37], [40]. However, this age effect seems to depend on emotional characteristic of negative pictures presented in the same block (arousal level of negative pictures).

Our study also evaluated the emotional bias depending on the age group and the considered contextual block. In the contextual block of high-arousal negative stimuli, given the relative arousal levels of emotional stimuli, a negativity bias was rationally observed in young adults [34], with an higher LPP amplitude and higher performances for high-arousal negative scenes rather than for positive and neutral scenes. This result attests that perceptual processing of high-arousal negative stimuli is particularly promoted compared to positive stimuli (with a lower arousal level) and neutral stimuli. From an evolutionary stand of point, events that threaten life, such as our high-arousal negative stimuli, would have a greater adaptive and motivational value than any other type of stimuli [63], [64]. In this situation, the motivational system would focus and favor attention to negative stimuli, crucial for adaptation and survival [65], [66] and would contribute to the facilitation of their perceptual processing, compared with other stimuli, as attested by amplitude variations of the LPP in our study and in the literature [34]. For high arousal stimuli, such as high-arousal negative stimuli, this facilitation would involve more automatic attentional processes [19], [67]. In young adults, this would also be the case for positive stimuli compared to neutral stimuli, which induced higher LPP amplitude and higher performance than neutral stimuli, in accordance with the literature [3].

In this contextual block of high-arousal negative stimuli, a negativity bias was also observed at the brain activity level in older adults, in agreement with previous behavioral studies [39], [41]. Indeed, the LPP amplitude measured for high-arousal negative stimuli was significantly higher than for positive and neutral stimuli, despite the fact that the LPP amplitude for negative stimuli was significantly reduced in older adults compared to younger adults. Moreover, older adults did not show a significant difference in amplitude of the LPP between the positive and neutral stimuli. This second result seems to differ from the one observed in young adults, although LPP amplitude induced by each of these two stimuli categories did not significantly vary between the two age groups, in this contextual block of high-arousal negative.

Taken together, these results in younger and older adults confirm the literature suggesting that the prevalence of high-arousal negative stimuli relative to the other stimuli, remains relatively well preserved in older people [39], [41] with, however, a possible contextual effect of these stimuli with age on relative processing of positive and neutral stimuli. According to model SAVI, internal homeostatic balance is restored later in older adults than in younger adults following the processing of emotional stimuli [37]. In our study, we may assume that this temporal delay in restoration of homeostatic balance with age would likely be longer in the contextual block of high arousal negative stimuli, due to processing of these negative stimuli. Consequently, it could partly justify the lack of distinction observed between positive and neutral stimuli, in this context, with older adults. However, this hypothesis cannot be confirmed here and should be explored in future work.

In the contextual block of low-arousal negative stimuli, results suggest differences in emotional preference between the two age groups. In young adults, a negativity bias, attested by LPP amplitude, was observed despite the fact that the arousal level of negative stimuli in this contextual block was significantly rated lower than the one of positive stimuli at the subjective level. This result, coupled with the previously observed one for the same age group, in other contextual block, suggests that young adults always promote a negativity bias even when the arousal level of negative stimuli is relatively low.

Moreover, in the contextual block of low-arousal negative stimuli, we noted that the LPP amplitudes for positive and neutral stimuli were statistically similar in young adults. This result seems to differ from the previously observed one, for the same age and in other contextual block, although no significant modulation of LPP amplitude was noted in young adults, for positive and neutral stimuli between the two blocks (i.e. with high-arousal or low-arousal negative stimuli). It could possibly be related to a specific, emotional regulation, in the context of low-arousal negative stimuli, this in favor of a higher attention for negative emotion rather than other emotion (negativity bias). However, this hypothesis cannot be confirmed here and should be explored in future work.

In older adults, only an emotional bias (without distinction of negative and positive stimuli) was noted in the contextual block of low-arousal negative stimuli, with a higher LPP amplitude for negative and positive stimuli compared to neutral stimuli. This kind of result was previously observed in the three EEG studies on the LPP amplitude using negative and positive stimuli with arousal levels close to those used here [30]–[32]. Based on previous behavioral studies [19], [38], [40] and the model SAVI, we assumed that the absence of negativity bias in older adults, in the contextual block of low-arousal negative stimuli, may be associated with a particular decrease in responses to low-arousal negative stimuli included in this block. A significant amplitude reduction of the LPP for negative stimuli, was effectively observed between the two age groups. But, it was independent of the two arousal levels of negative stimuli. However, we noted that the low-arousal negative stimuli induced a LPP amplitude significantly lower than the one induced by the high-arousal negative stimuli, regardless of age. In addition, we showed no significant age effect on processing of positive stimuli in the two contextual blocks of negative stimuli. Given these two results, the significant activity reduction observed with age for negative stimuli, may partly justify the absence of negativity bias in older adults and in the contextual block of low-arousal negative stimuli. Moreover, in this contextual block, older adults seem to favor an emotional bias by specifically reducing the activity associated with neutral situations. This last result suggests that the age effect on processing of neutral stimuli has to be more considered in the understanding of age-related positivity effect.

The age-related effects on cerebral processing that we observed, appeared without consequences on emotional categorization performances (performed during the experiment) as well as on subjective rating of the pictures' arousal level (reported after the experiment). Indeed, no significant difference was observed between the two age groups for behavioral and subjective data. This dissociation is in agreement with previous studies in ERP [32], [68] using natural scenes (IAPS) as stimuli. It suggests that age does not affect the conscious emotional appraisal of pictures (in terms of hedonic valence, discrete emotions and arousal level) and only modulates their cerebral processing. However, other purely behavioral studies go against this hypothesis and report changes with age in the subjective rating of emotional scenes [37], [38], [41]. Various methodological choices in this study may have contributed to the lack of age effect on our behavioral and subjective measures. Firstly, as in many studies in EEG and fMRI, the emotional categorization of pictures was performed at the end of each picture and not during the presentation of pictures. Participants had thus the time to re-evaluate pictures in a more rational manner. Moreover, this categorization involved the identification of discrete emotions (fear or well-being or no emotion), whereas the emotional valence (pleasantness versus unpleasantness) is classically evaluated in other studies. As for the subjective rating of pictures' arousal level (performed after the EEG experiment), it is possible that an habituation effect appeared since participants saw the images for the second time. In addition, pictures were not rated in the same configuration as the EEG experiment (by considering the two contexts).

Based on cerebral activity data, our results support the idea that age impacts the processing of negative information, regardless of its arousal level. However, the relative processing of different arousal levels of negative stimuli appears similar between the two age groups. The processing of positive information is preserved with age and is not contextually impacted by the arousal level of negative stimuli, regardless of age. As for the processing of neutral stimuli, we show it can be modulated with age, particularly in a context of low-arousal negative stimuli. Our study suggests that all these age-related effects may justify emotional bias differences observed with aging, in literature. These results should be confirmed with other subjects, in both age groups. Moreover, it should be noted that it is not excluded that the processing of positive stimuli (rather than negative stimuli) may be modulated with age, depending on their arousal level. We may also assume that this arousal level of positive stimuli can contextually influence the processing of negative (and neutral) stimuli as well and contribute to the changes in emotional preferences with age. These hypotheses should be explored in future work. In conclusion, our study confirms the interest of taking into account, both the properties of emotional stimuli and the reciprocal influences between these stimuli, for a better understanding of the positivity effect with age.

## Supporting Information

Table S1 **The picture ratings obtained during the pretests and the experiment for each age group.**
(DOC)
