# Supplementary material for: Brain Processing of Emotional Scenes in Aging: Effect of Arousal and Affective Context
Source: PLoS One. 2014 Jun 16;9(6):e99523. doi: 10.1371/journal.pone.0099523 (PMC4059675; doi:10.1371/journal.pone.0099523)
Supplement: Table S1 — The picture ratings obtained during the pretests and the experiment for each age group. (DOC) [file pone.0099523.s001.doc]

Table S1: The picture ratings by age group obtained during the pretests and the experiment.

|  | ***PRETEST 1*** | ***PRETEST 2*** | ***PRETEST 2*** | ***EXPERIMENT*** | ***EXPERIMENT*** |
| --- | --- | --- | --- | --- | --- |
|  | **Young adults (34)** | **Young adults (19)** | **Older adults (17)** | **Young adults (11)** | **Older adults (11)** |
| **Description (IAPS Number)** | ***Arousal (±SE[[1]](#footnote-2))*** | ***Valence (±SE)*** | ***Valence (±SE)*** | ***Arousal (±SE)*** | ***Arousal (±SE)*** |
| **HIGH-AROUSAL NEGATIVE** |  |  |  |  |  |
| **Animal (12)** |  |  |  |  |  |
| agressiv dog (IAPS:1300) | 7,25 (±1,81) | 1,52 (±2) | 1,51 (±2,05) | 7,5 (±1,84) | 7,72 (±2,43) |
| crocodil | 7,08 (±1,79) | 1,97 (±1,89) | 2,27 (±1,82) | 6,65 (±1,83) | 6,79 (±2,31) |
| shark | 7,81 (±1,87) | 1,67 (±1,95) | 1,78 (±1,96) | 7,93 (±1,89) | 8,09 (±2,53) |
| shark | 7,28 (±1,98) | 1,68 (±1,85) | 2,13 (±1,86) | 7,16 (±1,91) | 7,81 (±2,51) |
| shark | 7,59 (±1,88) | 1,85 (±1,85) | 2,6 (±1,75) | 6,72 (±1,85) | 6,37 (±2,3) |
| shark | 8,2 (±1,98) | 1,45 (±1,99) | 2,59 (±1,7) | 7,6 (±1,84) | 6,55 (±2,26) |
| shark | 7,86 (±1,78) | 1,75 (±1,88) | 2,18 (±1,8) | 7,76 (±1,9) | 8,08 (±2,54) |
| snake (IAPS:1050) | 8,43 (±2,07) | 1,97 (±1,89) | 2,27 (±1,77) | 7,35 (±1,96) | 8,15 (±2,54) |
| snake (IAPS:1052) | 7,61 (±1,77) | 2,18 (±1,92) | 1,62 (±2,04) | 6,46 (±1,85) | 6,91 (±2,36) |
| snake | 7,52 (±1,88) | 1,57 (±1,98) | 1,93 (±2) | 7,07 (±1,95) | 6,83 (±2,4) |
| snake | 7,24 (±1,79) | 2,29 (±1,92) | 2,49 (±1,83) | 5,8 (±1,82) | 7 (±2,38) |
| spider | 7,35 (±1,87) | 1,57 (±2,01) | 2,06 (±1,95) | 7,59 (±1,9) | 6,16 (±2,32) |
|  |  |  |  |  |  |
| **Environment/Landscape (10)** |  |  |  |  |  |
| bomb explosion | 7,18 (±1,95) | 1,61 (±2) | 1,75 (±2,01) | 7,14 (±1,81) | 7,18 (±2,35) |
| fiery cloud | 7,08 (±1,66) | 1,95 (±1,97) | 1,84 (±1,91) | 6,75 (±1,92) | 5,91 (±2,26) |
| lava (IAPS:5940) | 7,23 (±1,88) | 2,3 (±1,85) | 2,61 (±1,78) | 6,8 (±1,9) | 7,04 (±2,33) |
| nuclear blast | 7,8 (±1,84) | 2,15 (±1,82) | 1,85 (±1,94) | 8,03 (±1,96) | 7,46 (±2,46) |
| tornado | 7,12 (±1,89) | 1,91 (±1,99) | 2,5 (±1,75) | 6,29 (±1,87) | 6,97 (±2,38) |
| tornado | 7,82 (±1,87) | 1,7 (±2) | 2,24 (±1,83) | 6,48 (±1,87) | 6,5 (±2,29) |
| tsunami with people | 7,07 (±1,67) | 2,05 (±1,93) | 2,67 (±1,85) | 5,68 (±1,82) | 5,93 (±2,33) |
| wildfire | 7,32 (±1,9) | 1,85 (±1,98) | 2,55 (±1,76) | 6,53 (±1,79) | 6,28 (±2,23) |
| wildfire | 7,72 (±1,99) | 1,8 (±1,98) | 1,69 (±1,97) | 7,19 (±1,94) | 7,21 (±2,36) |
| wildfire | 7,23 (±1,91) | 1,97 (±1,93) | 2,56 (±1,64) | 5,88 (±1,75) | 7,63 (±2,44) |
|  |  |  |  |  |  |
| **Human treathening (8)** |  |  |  |  |  |
| child with a gun (IAPS:2811) | 7,62 (±1,92) | 1,59 (±1,95) | 1,3 (±2,1) | 6,74 (±1,82) | 7,74 (±2,36) |
| hand with a gun (IAPS:6260) | 7,92 (±2) | 1,81 (±1,97) | 1,84 (±1,75) | 6,83 (±1,88) | 8,05 (±2,37) |
| man brutalizing an hostage | 7,77 (±1,95) | 1,47 (±2) | 1,31 (±2,06) | 7,23 (±1,9) | 8,23 (±2,49) |
| man with a gun | 7,07 (±1,79) | 1,7 (±1,97) | 1,68 (±1,97) | 6,67 (±1,91) | 7,81 (±2,36) |
| man with a gun (IAPS:6250) | 7,4 (±1,76) | 1,68 (±1,97) | 1,45 (±2,08) | 6,78 (±1,81) | 8,03 (±2,36) |
| man with a gun | 7,08 (±1,77) | 2,28 (±1,93) | 1,94 (±1,9) | 5,68 (±1,83) | 6,87 (±2,17) |
| robber with a gun | 7,35 (±1,85) | 1,77 (±1,94) | 1,5 (±2,02) | 6,79 (±1,9) | 8 (±2,39) |
| robber with a knife (IAPS:6510) | 7,4 (±1,72) | 1,52 (±1,94) | 1,71 (±1,93) | 6,83 (±1,86) | 7,76 (±2,35) |
|  |  |  |  |  |  |
| **LOW-AROUSAL NEGATIVE** |  |  |  |  |  |
| **Animal (12)** |  |  |  |  |  |
| agressive bufle | 4,44 (±1,62) | 3,55 (±1,79) | 4,8 (±1,79) | 3,67 (±1,7) | 4,28 (±2,27) |
| agressive dog | 4,23 (±1,52) | 2,87 (±1,82) | 2,94 (±1,84) | 3,87 (±1,79) | 5,82 (±2,22) |
| agressive dog in a car (IAPS:1303) | 4,71 (±1,83) | 2,68 (±1,96) | 2,63 (±1,88) | 5,71 (±1,83) | 5,78 (±2,08) |
| agressive dog with a soldier | 4,92 (±1,64) | 2,57 (±1,92) | 2,21 (±1,93) | 5,64 (±1,8) | 6,39 (±2,35) |
| agressive eagle (IAPS:1560) | 3,89 (±1,77) | 3,1 (±1,85) | 5,17 (±1,75) | 3,42 (±1,79) | 4,6 (±2,23) |
| agressive eagle | 3,17 (±1,57) | 4,45 (±1,8) | 4,94 (±1,77) | 2,18 (±1,76) | 3,15 (±2,22) |
| cockroach | 4,37 (±1,57) | 2,43 (±1,91) | 3,26 (±1,8) | 4,91 (±1,75) | 4,92 (±2,25) |
| panther | 3,54 (±1,3) | 3,81 (±1,79) | 4,83 (±1,75) | 3,91 (±1,5) | 4,47 (±2,23) |
| mole-rat | 3,89 (±1,6) | 2,03 (±1,88) | 2,53 (±1,89) | 4,42 (±1,71) | 5,95 (±2,35) |
| aggressive meerkat | 3,57 (±1,77) | 2,6 (±1,88) | 3,26 (±1,89) | 3,5 (±1,79) | 4,38 (±2,24) |
| tiger | 4,19 (±1,38) | 3,07 (±1,79) | 2,88 (±1,77) | 4,74 (±1,89) | 5,76 (±2,29) |
| tiger (IAPS:1650) | 3,36 (±1,53) | 4,5 (±1,82) | 4,48 (±1,73) | 3,33 (±1,72) | 4,31 (±2,21) |
|  |  |  |  |  |  |
| **Environment/Landscape (10)** |  |  |  |  |  |
| boat in a tempest | 4,79 (±1,56) | 2,85 (±1,79) | 3,59 (±1,83) | 4,93 (±1,7) | 4,17 (±2,29) |
| car in fire | 4,88 (±1,62) | 2,14 (±1,9) | 1,87 (±1,98) | 5,12 (±1,85) | 6,63 (±2,26) |
| fireman against a wildfire | 4,42 (±1,65) | 2,25 (±1,96) | 2,23 (±1,92) | 5,61 (±1,82) | 6,64 (±2,35) |
| overflood river | 3,43 (±1,69) | 3,29 (±1,88) | 5,02 (±1,74) | 1,96 (±1,81) | 4 (±2,11) |
| people in flood | 4,41 (±1,73) | 2,13 (±1,91) | 2,44 (±1,89) | 4,37 (±1,89) | 7,04 (±2,33) |
| water wave on a dike | 4,53 (±1,54) | 3,62 (±1,81) | 3,89 (±1,71) | 3,79 (±1,67) | 4,2 (±2,18) |
| water wave on a dike | 3,75 (±1,35) | 3,27 (±1,85) | 4,21 (±1,81) | 4,34 (±1,72) | 3,71 (±2,22) |
| syringe | 4,41 (±1,54) | 3,58 (±1,75) | 3,13 (±1,75) | 4,33 (±1,57) | 4,4 (±2,04) |
| tornado | 4,28 (±1,66) | 2,56 (±1,88) | 2,76 (±1,88) | 6,12 (±1,82) | 4,54 (±2,17) |
| tropical tempest | 4,35 (±1,35) | 2,62 (±1,85) | 2,91 (±1,81) | 5,87 (±1,82) | 5,46 (±2,24) |
|  |  |  |  |  |  |
| **Human treathening (8)** |  |  |  |  |  |
| aggressive face | 3,94 (±1,78) | 2,4 (±1,92) | 2,39 (±1,88) | 4,05 (±1,72) | 6,39 (±2,39) |
| angry faces | 3,5 (±1,78) | 2,97 (±1,88) | 2,66 (±1,88) | 3,47 (±1,88) | 5,52 (±2,38) |
| angry faces | 3,58 (±1,85) | 2,88 (±1,81) | 2,34 (±1,91) | 3,21 (±1,87) | 5,71 (±2,33) |
| angry man | 3,33 (±1,78) | 2,55 (±1,93) | 2,06 (±1,97) | 3,61 (±1,82) | 6,66 (±2,33) |
| a hand with a bomb (IAPS:2692) | 4,96 (±1,6) | 1,97 (±1,89) | 3,67 (±1,77) | 5,75 (±1,8) | 5,24 (±2,19) |
| man with a gun | 4,74 (±1,65) | 2,46 (±1,82) | 2,34 (±1,89) | 5,8 (±1,77) | 7,63 (±2,32) |
| soldier with gun | 3,32 (±1,5) | 2,39 (±1,9) | 3,02 (±1,83) | 5,82 (±1,73) | 3,23 (±2,28) |
| soldier with gun | 4,68 (±1,71) | 2,39 (±1,9) | 1,64 (±2,04) | 5,39 (±1,8) | 7,01 (±2,32) |
|  |  |  |  |  |  |
| **NEUTRAL** |  |  |  |  |  |
| **Animal (3)** |  |  |  |  |  |
| bird on a house | 1,07 (±1,85) | 4,6 (±1,89) | 5,18 (±2,01) | 1,22 (±1,97) | 2,57 (±2,34) |
| bird in a tree | 1,3 (±2,04) | 4,34 (±1,87) | 5,88 (±1,9) | 1,02 (±1,87) | 3,97 (±2,3) |
| bird in a tree | 0,86 (±1,8) | 5,58 (±1,84) | 5,82 (±1,95) | 1,23 (±1,95) | 2,92 (±2,35) |
|  |  |  |  |  |  |
| **Environment/Landscape (16)** |  |  |  |  |  |
| bush | 0,8 (±2,12) | 4,51 (±1,97) | 4,85 (±2,03) | 1,06 (±1,92) | 2,61 (±2,34) |
| bushes | 0,99 (±2,2) | 4,58 (±2,03) | 5,16 (±2,02) | 1,12 (±1,98) | 1,73 (±2,42) |
| bushes and trees | 0,74 (±2,1) | 4,71 (±1,91) | 5,08 (±1,93) | 1,14 (±1,85) | 3,58 (±2,35) |
| bycicle parking | 0,54 (±1,91) | 4,76 (±2,01) | 4,77 (±1,99) | 1,49 (±1,9) | 1,13 (±2,67) |
| city road | 0,74 (±1,79) | 4,39 (±1,96) | 5,02 (±1,89) | 1,33 (±2,01) | 1,02 (±2,56) |
| forest | 0,63 (±2) | 4,8 (±1,92) | 5,41 (±1,97) | 1,12 (±2) | 3,17 (±2,32) |
| hall | 0,6 (±2) | 4,78 (±1,99) | 4,46 (±1,89) | 1,14 (±1,96) | 1,42 (±2,59) |
| hangar | 1,37 (±2,06) | 4,57 (±2,04) | 4,72 (±1,97) | 1,24 (±2,03) | 1,04 (±2,49) |
| house | 0,95 (±1,86) | 4,37 (±1,89) | 4,9 (±2,02) | 1,34 (±1,96) | 1,39 (±2,57) |
| opened briefcase | 1,26 (±1,9) | 4,92 (±1,98) | 4,78 (±2) | 1,11 (±2,08) | 1,19 (±2,62) |
| outside stairs | 0,61 (±1,74) | 4,85 (±1,97) | 4,99 (±2,04) | 1,06 (±1,98) | 1,06 (±2,66) |
| rail crossing | 1,25 (±1,94) | 4,45 (±1,95) | 4,71 (±2,02) | 1,28 (±2,03) | 1,06 (±2,66) |
| road in a plain | 0,78 (±1,8) | 4,78 (±1,98) | 5,59 (±1,91) | 1,75 (±1,91) | 1,49 (±2,53) |
| road bordered by a railway | 0,73 (±1,68) | 4,69 (±1,91) | 4,84 (±2,04) | 1,2 (±2) | 1,04 (±2,6) |
| sand dunes | 1,2 (±1,5) | 4,72 (±1,92) | 5,52 (±1,97) | 1,89 (±1,8) | 3,28 (±2,39) |
| stone in grass | 0,97 (±1,84) | 4,12 (±1,92) | 4,77 (±1,99) | 1,28 (±1,99) | 1,44 (±2,27) |
|  |  |  |  |  |  |
| **Inside home (10)** |  |  |  |  |  |
| bathroom | 1,13 (±2) | 4,07 (±1,97) | 4,91 (±2,05) | 1,31 (±1,96) | 1,1 (±2,69) |
| hall | 0,83 (±2,12) | 4,66 (±1,93) | 4,69 (±2) | 1,65 (±1,84) | 1,27 (±2,62) |
| inside a train | 0,88 (±2,09) | 4,85 (±1,97) | 4,76 (±1,92) | 1,01 (±2,05) | 1,27 (±2,59) |
| stairs | 0,89 (±1,82) | 4,56 (±1,95) | 5 (±1,97) | 1,8 (±1,91) | 1,28 (±2,63) |
| brick | 1,12 (±2,15) | 5 (±1,98) | 4,85 (±1,95) | 1,05 (±1,99) | 1,07 (±2,62) |
| door of a house | 1,2 (±2,05) | 4,97 (±2,07) | 4,83 (±2,04) | 1,31 (±1,95) | 1,03 (±2,7) |
| stairs | 0,65 (±2,08) | 4,25 (±1,94) | 4,75 (±2,01) | 1,48 (±1,97) | 1,06 (±2,67) |
| tiles | 1,18 (±2,12) | 4,33 (±1,91) | 4,79 (±2) | 1,33 (±1,95) | 1,04 (±2,64) |
| tiles | 0,67 (±2,02) | 3,91 (±1,86) | 5,36 (±1,92) | 1,14 (±2) | 1,67 (±2,34) |
| wall | 0,69 (±1,59) | 4,91 (±1,91) | 5,49 (±1,98) | 1,22 (±1,96) | 2,22 (±2,36) |
|  |  |  |  |  |  |
| **Object (19)** |  |  |  |  |  |
| adaptator | 0,82 (±2,16) | 5,11 (±2,03) | 4,74 (±1,99) | 1,06 (±2,04) | 1,15 (±2,68) |
| bag on a chair | 0,91 (±2,01) | 4,77 (±2,06) | 4,87 (±2,03) | 1 (±2,11) | 1,11 (±2,64) |
| basin | 0,9 (±1,99) | 4,96 (±1,91) | 4,78 (±2,02) | 1,51 (±1,94) | 1,24 (±2,67) |
| bench | 0,79 (±2,13) | 4,75 (±1,9) | 4,93 (±1,98) | 1,25 (±1,9) | 1,05 (±2,69) |
| carpet | 0,73 (±1,95) | 5,12 (±2,08) | 4,88 (±2) | 1,34 (±1,97) | 1,06 (±2,69) |
| room with columns (IAPS:7161) | 0,88 (±2,06) | 4,35 (±1,95) | 4,96 (±2,04) | 1,1 (±2,03) | 1,02 (±2,68) |
| jug | 0,93 (±2,07) | 4,56 (±1,93) | 4,99 (±2,03) | 1,07 (±2,01) | 1,1 (±2,56) |
| keys | 1,07 (±2,05) | 4,97 (±1,98) | 5,14 (±1,94) | 1,03 (±2) | 1,5 (±2,55) |
| keys | 0,96 (±2,09) | 4,91 (±2,05) | 4,87 (±2,05) | 1,07 (±2,06) | 1,33 (±2,6) |
| mug (IAPS:7009) | 0,62 (±1,78) | 5,29 (±2,07) | 4,95 (±2) | 1,14 (±2,04) | 1,66 (±2,51) |
| a woodworking tool | 0,93 (±2,15) | 4,97 (±1,93) | 5,2 (±1,95) | 1,39 (±1,92) | 1,12 (±2,55) |
| plate | 0,63 (±2,1) | 4,7 (±2) | 5,06 (±2,03) | 1,16 (±2,05) | 1,18 (±2,58) |
| ruler and crayon | 0,74 (±1,99) | 5,12 (±1,96) | 5,23 (±2,06) | 1,62 (±1,83) | 1,11 (±2,65) |
| sandals | 0,96 (±1,98) | 3,91 (±1,94) | 4,19 (±1,9) | 1,58 (±1,9) | 1,21 (±2,63) |
| stool | 0,56 (±2,02) | 4,82 (±1,99) | 4,94 (±2,08) | 1,07 (±1,97) | 1,11 (±2,67) |
| table and chairs | 0,9 (±2,03) | 4,85 (±2,01) | 4,81 (±2,03) | 1,46 (±1,96) | 1,1 (±2,68) |
| table, chair, pen, agenda | 0,59 (±1,88) | 4,87 (±2,07) | 4,89 (±1,97) | 1,05 (±2,08) | 1,09 (±2,59) |
| bench vice | 1,25 (±1,94) | 4,61 (±1,96) | 4,93 (±2,06) | 1,28 (±2) | 1,1 (±2,63) |
| wooden crate | 1,12 (±2,18) | 4,82 (±1,96) | 4,81 (±2,05) | 1,11 (±2,04) | 1,08 (±2,64) |
|  |  |  |  |  |  |
| **Object in a context (10)** |  |  |  |  |  |
| cut branches | 0,93 (±2,14) | 4,07 (±1,89) | 4,86 (±2,02) | 1,25 (±1,88) | 1,38 (±2,48) |
| cut branches | 0,88 (±2,05) | 4,58 (±1,93) | 5,01 (±1,98) | 1,04 (±1,74) | 2,05 (±2,5) |
| fire hydrant | 1,19 (±1,97) | 4,95 (±2,12) | 4,91 (±2,05) | 1,06 (±2,09) | 1,05 (±2,69) |
| floor lamp | 1,02 (±1,87) | 4,69 (±1,97) | 4,87 (±2,03) | 1,05 (±2,05) | 1,13 (±2,57) |
| office | 1,29 (±1,5) | 5,31 (±1,95) | 5,1 (±1,98) | 1,53 (±1,83) | 1,03 (±2,66) |
| radiator | 0,99 (±2,08) | 4,77 (±1,93) | 4,69 (±1,96) | 1,1 (±2,07) | 1,13 (±2,67) |
| security barrier | 1,04 (±2) | 4,95 (±1,98) | 4,88 (±2,09) | 1,27 (±1,97) | 1,06 (±2,69) |
| table with chairs | 0,54 (±2,1) | 4,99 (±1,94) | 5,05 (±1,99) | 1,42 (±2,02) | 1,62 (±2,49) |
| traffic sign | 0,92 (±2,22) | 4,73 (±2,01) | 4,52 (±1,9) | 1,02 (±1,92) | 1,26 (±2,61) |
| train at a station platform | 1 (±1,88) | 5,05 (±1,91) | 5 (±2,03) | 1,06 (±2,06) | 1,55 (±2,42) |
|  |  |  |  |  |  |
| **People (2)** |  |  |  |  |  |
| people in a train | 0,94 (±1,93) | 4,78 (±2) | 4,73 (±1,97) | 1,32 (±2) | 1,04 (±2,66) |
| man which repairs antennas | 1,09 (±2,15) | 4,28 (±1,87) | 4,48 (±1,9) | 1,2 (±2,04) | 1,68 (±2,41) |
|  |  |  |  |  |  |
| **POSITIVE** |  |  |  |  |  |
| **Animal (17)** |  |  |  |  |  |
| a man with a giraffe | 2,88 (±1,73) | 6,7 (±1,98) | 6,85 (±1,89) | 4,57 (±1,75) | 5,63 (±2,32) |
| camels | 3,02 (±1,71) | 6,68 (±2,01) | 7,19 (±1,93) | 5,38 (±1,81) | 6,35 (±2,43) |
| camels | 2,48 (±1,67) | 6,23 (±1,92) | 6,42 (±1,88) | 4,14 (±1,73) | 5,15 (±2,37) |
| cat | 3,56 (±1,66) | 7,21 (±1,68) | 6,44 (±1,78) | 4,23 (±1,6) | 5,75 (±2,28) |
| cat | 3,42 (±1,74) | 6,91 (±1,72) | 6,57 (±1,76) | 3,86 (±1,57) | 5,85 (±2,29) |
| fishes in an underwater background | 2,79 (±1,65) | 7,18 (±1,86) | 7,36 (±1,91) | 5,79 (±1,92) | 5,89 (±2,39) |
| fishes in an underwater background | 3,29 (±1,62) | 7,45 (±1,87) | 7,65 (±1,95) | 5,6 (±1,8) | 7,15 (±2,49) |
| dolphin | 3,68 (±1,58) | 7,19 (±1,83) | 6,74 (±1,91) | 5,02 (±1,86) | 4,97 (±2,19) |
| dolphin | 3,3 (±1,69) | 6,85 (±1,84) | 7,02 (±1,93) | 5,85 (±1,85) | 3,72 (±2,11) |
| dolphin | 2,85 (±1,62) | 7,1 (±1,78) | 7,47 (±1,98) | 5,94 (±1,81) | 4,8 (±2,16) |
| dolphin | 3,54 (±1,7) | 7,21 (±1,88) | 6,96 (±1,98) | 5,55 (±1,78) | 4,74 (±2,09) |
| kitten | 3,02 (±1,74) | 6,94 (±1,78) | 6,69 (±1,91) | 4,06 (±1,43) | 5,85 (±2,26) |
| kitten | 3,96 (±1,78) | 6,91 (±1,69) | 6,82 (±1,88) | 4,3 (±1,52) | 6,26 (±2,39) |
| little dog | 2,73 (±1,69) | 7,7 (±1,83) | 7,29 (±1,98) | 4,94 (±1,74) | 6,2 (±2,29) |
| penguin on the ice | 2,4 (±1,81) | 6,66 (±1,89) | 7,19 (±1,95) | 5,28 (±1,8) | 4,9 (±2,27) |
| squirrel | 3,46 (±1,7) | 6,97 (±1,94) | 7,28 (±1,95) | 5,58 (±1,74) | 6,77 (±2,43) |
| turtle | 2,94 (±1,75) | 6,98 (±1,85) | 7,5 (±1,98) | 5,44 (±1,76) | 5,92 (±2,37) |
|  |  |  |  |  |  |
| **Environment/Landscape (26)** |  |  |  |  |  |
| aztec temple | 3,62 (±1,66) | 6,85 (±1,99) | 7,11 (±1,88) | 4,81 (±1,93) | 6,73 (±2,49) |
| beach | 3,19 (±1,27) | 7,58 (±1,85) | 6,9 (±1,84) | 4,56 (±1,72) | 5,78 (±2,26) |
| beach | 3,58 (±1,62) | 6,74 (±1,94) | 6,91 (±1,88) | 5,1 (±1,88) | 4,96 (±2,39) |
| beach | 5,05 (±1,79) | 7,82 (±1,95) | 7,79 (±1,95) | 6,11 (±1,84) | 6,74 (±2,39) |
| beach | 4,35 (±1,65) | 7,71 (±1,89) | 7,46 (±1,87) | 6,19 (±1,87) | 7,68 (±2,52) |
| beach | 4,39 (±1,79) | 7,55 (±1,96) | 7,23 (±1,82) | 5,03 (±1,87) | 7,48 (±2,54) |
| beach | 3,76 (±1,61) | 7,51 (±1,86) | 7,59 (±1,89) | 6,37 (±1,93) | 7,78 (±2,54) |
| beach | 5,13 (±1,74) | 7,57 (±1,83) | 7,38 (±1,88) | 6,68 (±1,92) | 7,13 (±2,44) |
| beach | 3,94 (±1,76) | 7,41 (±1,85) | 7,79 (±1,97) | 5,72 (±1,84) | 7,88 (±2,57) |
| island with beach | 5,5 (±1,85) | 8,25 (±1,97) | 7,54 (±1,83) | 6,91 (±1,88) | 7,63 (±2,48) |
| island | 2,95 (±1,81) | 7,64 (±1,95) | 7 (±1,85) | 5,81 (±1,86) | 6,78 (±2,49) |
| island | 3,04 (±1,68) | 7,17 (±1,86) | 7,14 (±1,94) | 4,1 (±1,93) | 4,91 (±2,3) |
| lake | 2,9 (±1,65) | 7,49 (±1,9) | 6,87 (±1,87) | 5,69 (±1,86) | 7,63 (±2,51) |
| lake in mountains | 3,51 (±1,8) | 6,85 (±1,83) | 7,41 (±1,86) | 6,6 (±1,91) | 7,25 (±2,46) |
| lake in mountains | 2,64 (±1,72) | 6,26 (±1,85) | 6,92 (±1,81) | 5,45 (±1,82) | 7,72 (±2,48) |
| mountain | 2,54 (±1,71) | 6,71 (±1,97) | 7,05 (±1,85) | 5,49 (±1,84) | 6,75 (±2,38) |
| mountain | 2,6 (±1,69) | 6,5 (±1,91) | 7,1 (±1,89) | 5,56 (±1,81) | 7,47 (±2,48) |
| river in mountains | 3 (±1,64) | 7,05 (±1,84) | 7,29 (±1,91) | 6,02 (±1,89) | 7,7 (±2,5) |
| mountain track | 2,79 (±1,7) | 6,68 (±1,83) | 6,98 (±1,86) | 5,8 (±1,83) | 7,28 (±2,47) |
| moutains | 2,52 (±1,67) | 7,39 (±1,89) | 7,26 (±1,94) | 5,86 (±1,84) | 6,63 (±2,36) |
| waterfall | 4,22 (±1,74) | 7,35 (±1,83) | 7,38 (±1,95) | 4,99 (±1,79) | 6,77 (±2,35) |
| road with a beach | 4,61 (±1,71) | 7,74 (±1,89) | 7,23 (±1,8) | 5,41 (±1,74) | 7,54 (±2,49) |
| seabed | 2,51 (±1,63) | 7,07 (±1,79) | 7,51 (±1,93) | 6,11 (±1,92) | 5,91 (±2,4) |
| sea | 5 (±1,73) | 8,2 (±1,94) | 7,4 (±1,87) | 6,76 (±1,87) | 8,02 (±2,6) |
| sea | 5,74 (±1,93) | 8,01 (±1,9) | 7,67 (±1,92) | 6,77 (±1,86) | 7,31 (±2,24) |
| sea | 4,91 (±1,72) | 7,45 (±1,81) | 7,35 (±1,9) | 6,64 (±1,9) | 8,08 (±2,58) |
|  |  |  |  |  |  |
| **Object in a context (4)** |  |  |  |  |  |
| sculptures | 3,77 (±1,75) | 6,6 (±1,91) | 7,59 (±1,94) | 5,57 (±1,79) | 5,94 (±2,34) |
| swimming pool | 3,43 (±1,7) | 7,51 (±1,89) | 6,77 (±1,83) | 3,53 (±1,81) | 4,98 (±2,37) |
| swimming pool | 3,76 (±1,73) | 7,22 (±1,77) | 7,56 (±1,87) | 4,05 (±1,75) | 6,44 (±2,52) |
| swimming pool | 4,48 (±1,68) | 7,95 (±1,93) | 6,88 (±1,84) | 3,98 (±1,66) | 5,94 (±2,31) |
|  |  |  |  |  |  |
| **Funny people in an environment (13)** |  |  |  |  |  |
| people watching a fireworks in a city | 3,09 (±1,69) | 7,33 (±1,86) | 7,23 (±1,79) | 3,94 (±1,76) | 5,64 (±2,35) |
| people watching a fireworks on the location of the Eiffel tower | 3,48 (±1,84) | 7,18 (±1,93) | 6,98 (±1,82) | 4,35 (±1,78) | 6,3 (±2,44) |
| people watching a fireworks on the location of the Eiffel tower | 3,1 (±1,65) | 7,11 (±1,95) | 6,82 (±1,78) | 4,66 (±1,78) | 6,4 (±2,51) |
| festivity | 2,43 (±1,48) | 5,94 (±1,64) | 4,77 (±1,72) | 2,86 (±1,77) | 1,36 (±2,29) |
| festivity | 2,47 (±1,29) | 5,77 (±1,7) | 5,61 (±1,79) | 2,63 (±1,85) | 2,1 (±2,22) |
| people playing volleyball | 3,1 (±1,63) | 6,87 (±1,9) | 5,83 (±1,9) | 3,99 (±1,86) | 4,16 (±2,38) |
| people who comes down a waterslide | 3 (±1,66) | 7,08 (±1,84) | 6,47 (±1,84) | 3,74 (±1,69) | 4,12 (±2,31) |
| people laughing on a waterslide | 2,84 (±1,66) | 6,78 (±1,76) | 6,84 (±1,88) | 2,97 (±1,68) | 6,03 (±2,3) |
| wedding party | 2,42 (±1,75) | 6,59 (±1,87) | 6,21 (±1,84) | 3,26 (±1,89) | 3,31 (±2,32) |
| baby kissing | 2,47 (±1,56) | 6,57 (±1,77) | 7,26 (±1,9) | 4,57 (±1,61) | 5,2 (±2,24) |
| a hand holding a chocolate box | 2,42 (±1,69) | 7,39 (±1,95) | 6,99 (±1,85) | 4,16 (±1,72) | 5,68 (±2,33) |
| people laughing in a diner | 2,43 (±1,63) | 7,02 (±1,88) | 6,67 (±1,89) | 3,41 (±1,91) | 5,77 (±2,44) |
| woman giving chocolate | 2,77 (±1,75) | 7,39 (±1,97) | 6,54 (±1,84) | 4,38 (±1,77) | 5,1 (±2,32) |

1. *SE = Standard Error* [↑](#footnote-ref-2)
